# New frontiers in domain-inspired radiomics and radiogenomics: increasing role of molecular diagnostics in CNS tumor classification and grading following WHO CNS-5 updates

**DOI:** 10.1186/s40644-024-00769-6

**Published:** 2024-10-07

**Authors:** Gagandeep Singh, Annie Singh, Joseph Bae, Sunil Manjila, Vadim Spektor, Prateek Prasanna, Angela Lignelli

**Affiliations:** 1 Neuroradiology Division, Columbia University Irving Medical Center, New York, NY USA; 2 Atal Bihari Vajpayee Institute of Medical Sciences, New Delhi, India; 3 Department of Biomedical Informatics, Stony Brook University, Stony Brook, USA; 4 Department of Neurological Surgery, Garden City Hospital, Garden City, MI USA

**Keywords:** Glioblastoma, Gliomas, Radiomics, Radiogenomics, Machine learning, Deep learning, CNS-5 classification updates

## Abstract

Gliomas and Glioblastomas represent a significant portion of central nervous system (CNS) tumors associated with high mortality rates and variable prognosis. In 2021, the World Health Organization (WHO) updated its Glioma classification criteria, most notably incorporating molecular markers including CDKN2A/B homozygous deletion, TERT promoter mutation, EGFR amplification, + 7/−10 chromosome copy number changes, and others into the grading and classification of adult and pediatric Gliomas. The inclusion of these markers and the corresponding introduction of new Glioma subtypes has allowed for more specific tailoring of clinical interventions and has inspired a new wave of Radiogenomic studies seeking to leverage medical imaging information to explore the diagnostic and prognostic implications of these new biomarkers. Radiomics, deep learning, and combined approaches have enabled the development of powerful computational tools for MRI analysis correlating imaging characteristics with various molecular biomarkers integrated into the updated WHO CNS-5 guidelines. Recent studies have leveraged these methods to accurately classify Gliomas in accordance with these updated molecular-based criteria based solely on non-invasive MRI, demonstrating the great promise of Radiogenomic tools. In this review, we explore the relative benefits and drawbacks of these computational frameworks and highlight the technical and clinical innovations presented by recent studies in the landscape of fast evolving molecular-based Glioma subtyping. Furthermore, the potential benefits and challenges of incorporating these tools into routine radiological workflows, aiming to enhance patient care and optimize clinical outcomes in the evolving field of CNS tumor management, have been highlighted.

## Background

Gliomas are defined as tumors arising from the neuroglial cells in the brain, while glioblastoma is a highly aggressive and advanced variant tumor with a relatively poor prognosis. Epidemiologically, gliomas occur among all ages, but are more often seen in adults with higher rates among males than females. The prognosis and outcome vary significantly depending on factors such as age at diagnosis, histomolecular characteristics, tumor grade, and the extent of surgical resection [1–3]. The 2021 World Health Organization (WHO) classification for central nervous system (CNS) tumors has introduced molecular parameters as key indicators for grading tumors and predicting patient outcomes across various tumor types [4]. This marks a significant shift from the previous paradigm, which primarily relied on histological grading. These revisions were primarily driven by advancements in understanding the molecular architecture of gliomas and the recognized influence of genetic mutations on the development of tumors and subsequent response to different treatment approaches.

The updated criteria now integrate genetic data for the categorization of certain tumor types, leading to the reclassification of particular entities. The classification of gliomas, glioneuronal tumors, and neuronal tumors has been expanded to include fourteen newly identified types. For an accurate diagnosis of certain types, like the diffuse high-grade pediatric type (H3-wildtype and IDH-wildtype) and diffuse low-grade glioma with MAPK pathway alterations, there is now a requirement to evaluate both histological features and molecular characteristics [4]. Molecular profiling has become a quintessential part of tumor classification and grading. For instance, IDH-mutant astrocytomas with CDKN2A/B homozygous deletion are now graded as high-grade astrocytomas (grade 4), and IDH-wildtype diffuse astrocytomas with TERT promoter mutation, EGFR amplification, or + 7/−10 copy number changes, can now be classified as molecular glioblastoma, even if their histological appearance suggests a lower grade. Notably, unlike earlier recommendations, CNS WHO grade is no longer solely determined by histological analysis, making tumor genotype a critical determinant for classification.

The update has also introduced new categories for pediatric-type gliomas to highlight their distinct nature. This includes both low-grade and high-grade types, each with specific molecular profiles that are key for classification and targeted treatment. Among high-grade tumors, diffuse midline gliomas with H3 K27 mutant have been updated to H3 K27 altered. This update acknowledges that alternative changes, such as EZHIP protein overexpression can now characterize this entity, expanding beyond the initially identified H3-K27 mutations. Diffuse pediatric-type high-grade gliomas, H3-wildtype, and IDH-wildtype are characterized as wildtype for both H3 and IDH gene families. Similar to many other CNS tumor types, this subtype requires a combination of molecular characterization and the integration of histopathological and molecular data for accurate diagnosis.

Furthermore, Ependymomas are now classified by combining histological, anatomical, and molecular data, which has led to the recognition of molecularly defined types in different brain regions. Medulloblastomas retain their four principal molecular groups (WNT, SHH, Group 3 and Group 4), but now feature additional subgroups identified through advanced profiling, which have diagnostic, prognostic, and therapeutic relevance.

In short, the updated WHO CNS-5 emphasizes genotyping and mutations across various brain tumor categories, signifying a shift toward a more molecularly oriented classification system. The granularity and diversity of this new classification system combined with its reliance on the collection of tissue samples for genetic marker testing has made it an attractive target for radiogenomic studies In the context of gliomas, radiogenomics promises to enable accurate molecular subtyping from non-invasive magnetic resonance imaging (MRI). Furthermore, due to the relative recency of the updated WHO CNS-5 guidelines, no review has comprehensively detailed recent radiogenomic studies tailored specifically to the biomarkers deemed significant by the new classification criteria. In this review we provide an overview of the different radiogenomic analysis frameworks employed in glioma radiogenomic studies before outlining recent radiogenomic studies for each of several key biomarkers. We further describe future opportunities for radiogenomic characterization of gliomas in the context of rapidly improving molecular profiling of these tumors and identify potential obstacles and unmet challenges.

## Radiomics and deep learning algorithms in Gliomas classification and grading

**Fig. 1:**
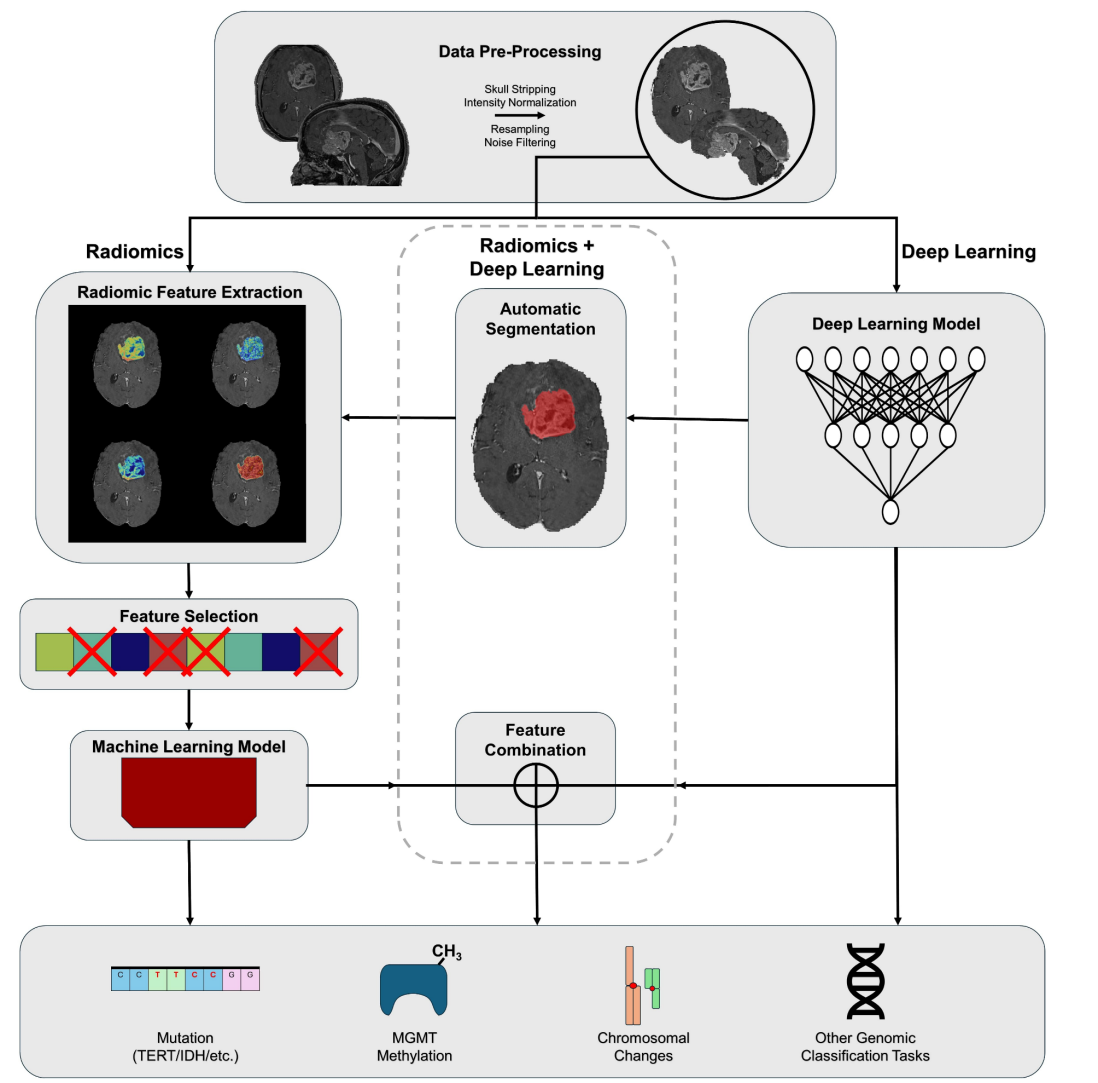
Radiomics, radiogenomics, and deep learning workflow

Radiogenomic analysis for Glioma molecular profiling has broadly fallen into three frameworks: (1) Radiomic studies, (2) Deep learning studies, and (3) combined Radiomic and Deep learning studies. Broadly, each of these frameworks consists of image acquisition, image pre-processing, and image feature extraction before completion of a downstream task such as genetic marker classification. For each approach, typical image pre-processing techniques include image resampling to uniform pixel spacing and slice thickness, skull-stripping, and some form of intensity normalization for MRI intensity values. Each of these techniques serves to reduce variability between scans from different patients, imaging devices, and institutions which allows for improved computational modelling of images. In some cases, registration to a reference or atlas brain MRI and noise reduction techniques including bias field correction might also be performed. Differences in radiomic, deep learning, and combined approaches to image feature extraction and the methodology employed for downstream classification will be described below. (Fig. 1)

### Radiomic analysis

Radiomic analysis of medical images has long been explored and leverages advanced mathematical filters to extract quantitative features from imaging modalities such as MRI, computed tomography (CT), positron emission tomography (PET), and ultrasound. In brain tumor imaging, these features are typically derived from a region of interest, commonly the tumor delineated on multi-parametric MRI scans, providing a comprehensive understanding of the three-dimensional (3D) tumor landscape inaccessible through biopsies or resected samples. Traditional radiomic filters capture attributes like texture and morphology, while emerging tools such as topological data analysis (TDA), including methods like persistent homology, offer topology-based radiomics descriptors, proven effective in diverse domains like breast cancer and vessel characterization in brain images. Because radiomic features all capture well-defined attributes within images (heterogeneity in texture, presence of spots/edges, existence of persistent linear patterns, etc.), radiomic analysis benefits from a large degree of interpretability, enabling understanding of which features of an image are significant for diagnosis, prognosis, or other imaging-based tasks.

Following feature extraction, radiomic studies commonly perform a feature selection step in order to more specifically identify which imaging features are likely to be of relevance for the downstream task. Feature selection is typically performed via identification of maximally relevant features and elimination of maximally redundant features, with one effective feature selection strategy aptly named “maximum relevance-minimum redundancy” [5]. Feature selection ensures that radiomic classifiers do not “overfit” on superfluous information that is not discriminative for the desired task. Selected radiomic features are typically input into a machine learning classifier such as a random forest or support vector machine model which is then trained for a specific task such as genetic marker identification. In general, model training is performed on radiomic features extracted from a subset of available patient data called the training split, and metrics are reported in studies based upon model performance on radiomic features extracted from an unseen test split of patient data.

### Deep learning analysis

Deep learning analysis of medical images leverages state-of-the-art neural networks to extract “deep” features or representations of images for classification and segmentation tasks. Unlike the pre-defined interpretability of radiomic features, deep features are learned by the neural network through iterative fitting of a model on input training images. These deep features often do not correspond to readily understood characteristics of an image, and are not easily understood/visualized when extracted from a trained neural network. For this reason, deep learning is often referred to as a “black box” approach with limited interpretability. However, because these features are learned directly from training a model on a specific imaging task, deep learning is often a more powerful form of image analysis with the capability to succeed in multiple medical imaging tasks. Deep learning models are often classified by the fundamental mechanism by which features are extracted from input medical images. Convolutional neural networks make use of learned mathematical filters to extract imaging features, whereas vision transformers calculate “attention” or similarity between different regions of an image. To combat the problem of overfitting, deep learning pipelines commonly employ data augmentation techniques such as flipping, adding small amounts of noise to, and rotating training images to prevent models from learning from these unimportant variations in input images. Feature extraction and the downstream task are typically performed in an integrated manner in deep learning analysis, in which a model learns both what features from an image to extract and how to model those features for a task such as genetic marker classification.

### Combined radiomic and deep learning analysis

Recent studies have also explored the combined use of radiomic and deep learning approaches for glioma medical image analysis. This integration of radiomic and deep learning features facilitates the intelligent application of more powerful deep learning methods while simultaneously retaining the interpretability of radiomic features, thereby bridging the gap between model performance and interpretability, a crucial step towards personalized and effective medical interventions. The emergence of combined deep learning and radiomics approaches signifies a paradigm shift in medical image analysis, offering a transition from ‘black box’ to ‘glass box’ quantitative methodologies. By incorporating radiomic and deep features, this hybrid approach enables clinicians to extract meaningful insights into disease progression and treatment response, beyond just model performance, thus enabling effective medical interventions. Typically, the combination of deep and radiomic features is undertaken by leveraging independent models trained to extract Radiomic and deep features via an ensembling of output probabilities for a given task [6, 7]. However, there have been multiple studies exploring more sophisticated integration of deep learning and radiomic approaches by augmenting radiomic features via deep learning [8], inputting radiomic features to a deep network [9] and using deep-learning based auto-segmentation of gliomas for radiomic feature extraction [6].

## Diagnostic markers

### Adult-type diffuse gliomas

#### IDH mutation

**Fig. 2:**
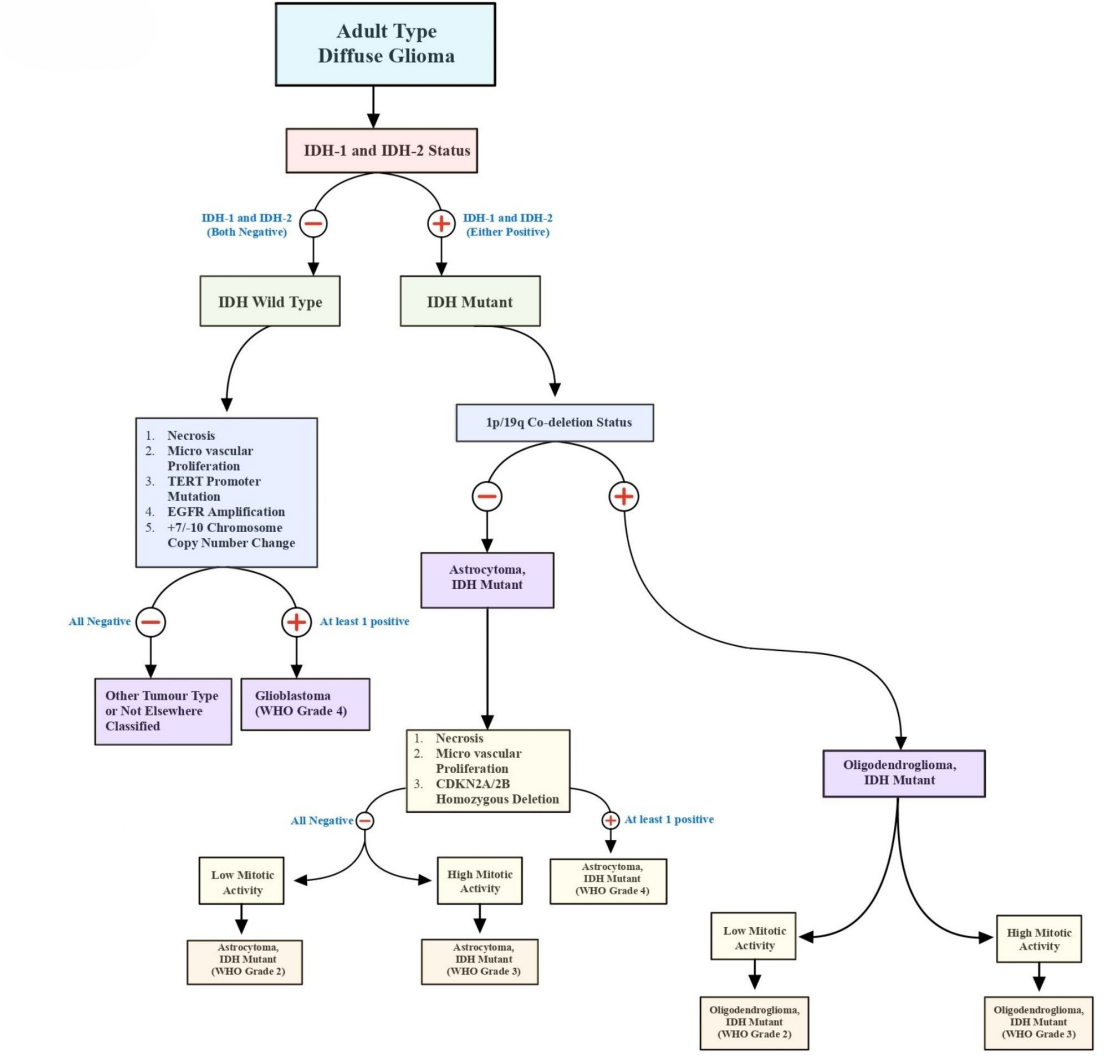
Adult type diffuse Glioma classification

Isocitrate Dehydrogenase (IDH) mutation is an important biomarker for gliomas, associated with better prognosis in comparison to its counterpart IDH wild-type tumors [10]. The importance of IDH mutation has been highlighted in the WHO 2021 CNS tumor classification update, wherein the IDH mutation is the basis of classifying adult-type diffuse gliomas. (Fig. 2) Recent advancements in the fields of radiomics, radiogenomics, and machine learning have prompted numerous studies exploring multiple directions for the prediction of IDH1 mutation status in gliomas through non-invasive methods.

The T2-FLAIR mismatch sign is a distinctive pattern observed in IDH-mutant diffuse astrocytomas, where the mass appears uniformly hyperintense on T2-weighted images but shows a relatively dark signal on FLAIR images, except for a bright peripheral ring [11]. The T2-FLAIR mismatch sign has high specificity but low sensitivity for IDH-mutant diffuse astrocytomas [12, 13]. Additionally, 2-Hydroglutarate (2HG) Magnetic Resonance Spectroscopy (MRS) has emerged as a valuable tool for identifying IDH mutations in Gliomas. Hirofumi et al. [14]. assessed the utility of 2HG-MRS for diagnosing IDH-mutant adult brainstem gliomas in ten patients with radiographically confirmed brainstem tumors who underwent 2HG-MRS followed by biopsy. Four patients had an H3K27M mutation, four had an IDH1 mutation, and two had neither mutation. The study found that a 2HG concentration ≥ 1.8 mM demonstrated 100% sensitivity and specificity for IDH-mutant brainstem gliomas.

Kasap et al. [15]. compared various MR sequences in predicting IDH mutation status, finding that the T1-w contrast-enhanced sequence was most optimal for predicting IDH mutations status using a radiomics-based model. He et al. [16]. proposed a “6-Step” general radiomics model to predict IDH mutation status in Glioma patients, proposing an SVM model trained on T2 + FLAIR sequences which yielded an AUC/accuracy/sensitivity/specificity of 0.873/ 0.876/ 0.875 /0.877. Hosseini et al [8]. used a deep learning-based data augmentation method (CTGAN) to synthesize 200 datasets from the training sets, which, when trained on T1-w post-contrast scans was able to achieve an AUC/accuracy/sensitivity of 0.93/0.92/1.00. Calabrese et al [6]. achieved an AUC of 0.96 in predicting IDH mutation status using a combined Convolutional Neural Network (CNN) and Radiomics model. Despite the success of these recent studies in predicting IDH mutation status, additional large-scale studies externally validated on data from multiple institutions are needed before these methods become a part of routine diagnostics.

#### TERT promoter mutation

TERT (Telomerase Reverse Transcriptase) promoter mutations are genetic alterations that can be found in many different cancers, including some CNS tumors. The new WHO CNS-5 classification has emphasized the role of TERTp mutations in the diagnosis and prognosis of CNS tumors. The presence of a TERT promoter mutation is considered a marker of high-grade malignancy, and its detection can lead to the upgrading of a CNS tumor to WHO Grade 4 [17]. Multiple groups have evaluated radiomics and radiogenomic features to determine TERTp mutation in gliomas. Fang et al. [18] analysed a total of 1293 radiomics features to train 10 predictive glioma models achieving an AUC/accuracy/sensitivity/specificity of 0.8446/0.7988/0.9355/0.6197. Zhang et al. [19] proposed a Deep Learning-Based Radiomics (DLR) signature for predicting TERT promoter mutations achieving an AUC of 0.890, exhibiting superior discriminative power compared to the Clinical Deep Learning Radiomics (CDLR) nomogram and clinical models in the validation cohort.

#### EGFR amplification

EGFR (Epidermal Growth Factor Receptor) expression, a cell membrane tyrosine kinase receptor, has been implicated in several mechanisms contributing to the abnormal and swift cell proliferation observed in various CNS tumors including glioblastomas. Among these pathways, elevated EGFR levels are frequently observed in primary GBMs, resulting from gene amplification, enhanced translation of the EGFR gene, or a combination of both processes. In the latest CNS-5 WHO classification update, the presence of EGFR amplification is sufficient for a tumor to be considered WHO Grade 4. Gupta et al. [20] reported that EGFR amplification is related to imaging features such as higher median relative cerebral blood volume (rCBV) and lower permeability-surface area product (PSR). Pasquini et al. [21] predicted EGFR amplification status using radiomic features extracted from rCBV and T2 images within CET ROI, rCBV demonstrated the highest performance with AUC/accuracy of 0.74/0.81 while the T2 sequence achieved AUC/accuracy of 0.741/0.778. Sohn et al. [22] yielded a poor performance in predicting EGFR amplification status (AUC = 0.743) compared to other markers such as IDH (AUC = 0.967) and MGMT (AUC = 0.761), suggesting the relative difficulty in evaluating EGFR status using structural MRI alone. The association between EGFR amplification may serve as a useful biomarker for poor prognosis in glioma patients [23]. Targeted therapies against EGFR and EGFRvIII has yielded mixed results [24] with some showing promising outcomes, however more evidence and work is needed to get a better understanding of EGFR/EGFRvIII pathway, its role in tumor prognosis, and ultimately better targeted therapeutics with improved brain penetration.

####  + 7/-10 Copy number change

The 2021 CNS tumor classification introduced trisomy 7 and monosomy 10 (+ 7/-10 copy number change) as a novel addition to classify glial tumors as WHO Grade-4. However, there are few studies exploring the relationship between imaging and radiomic features of + 7/-10 copy number change. One study performed by Calabrese et al. [6]. found a significant correlation between the prediction of chromosome 7/10 aneuploidy and the elongation of the enhancing tumor on imaging. The study also reported an optimal AUC/sensitivity/specificity of 0.93/0.90/0.88 for predicting + 7/-10 copy number change. The validation of these results remains challenging due to lack of extensive research on the diagnostic potential of + 7/-10 copy number change. Consequently, there is a pressing requirement for further comprehensive studies to address this gap in the literature.

#### 1p/19q co-deletion

The presence of 1p/19q codeletion is a significant diagnostic marker required for histomolecular diagnosis of oligodendroglioma [25, 26]. The recent WHO 2021 CNS-5 update now requires the determination of 1p/19q codeletion status for the final diagnosis of oligodendroglioma (IDH mutant, 1p/19q co-deleted). In lower-grade gliomas, immune cell infiltration into the tumor microenvironment is driven by cytokines and chemokines, which are encoded by 41 genes located on chromosome 1p/19q. When 1p/19q is co-deleted, the activity of these genes is diminished compared to gliomas without the deletion. This reduction leads to a weakened immune response, characterized by fewer immune cells infiltrating the tumor and decreased expression of genes involved in immune checkpoint regulation [27]. Therefore, accurately predicting the 1p/19q codeletion status in gliomas could play a significant role in predicting outcomes for patients with CNS tumors. Batchala et al. [13] achieved an accuracy = 86.3% for predicting the 1p/19q codeletion status, with tumor heterogeneity, frontal lobe location, and T2 susceptibility blooming to be significant predictors for 1p/19q-codeletion status. Kihira et al. [28] predicted the 1p/19q codeletion status with an AUC/sensitivity/specificity/accuracy of 0.85/0.779/0.828/0.803, using a radiomic model generated from combination of six texture features. The radiomic model yielded significantly improved sensitivity in predicting the 1p/19q codeletion status than T2-FLAIR mismatch sign and the clinical model.

### Pediatric-type diffuse high-grade gliomas

#### H3 K27-altered

**Fig. 3:**
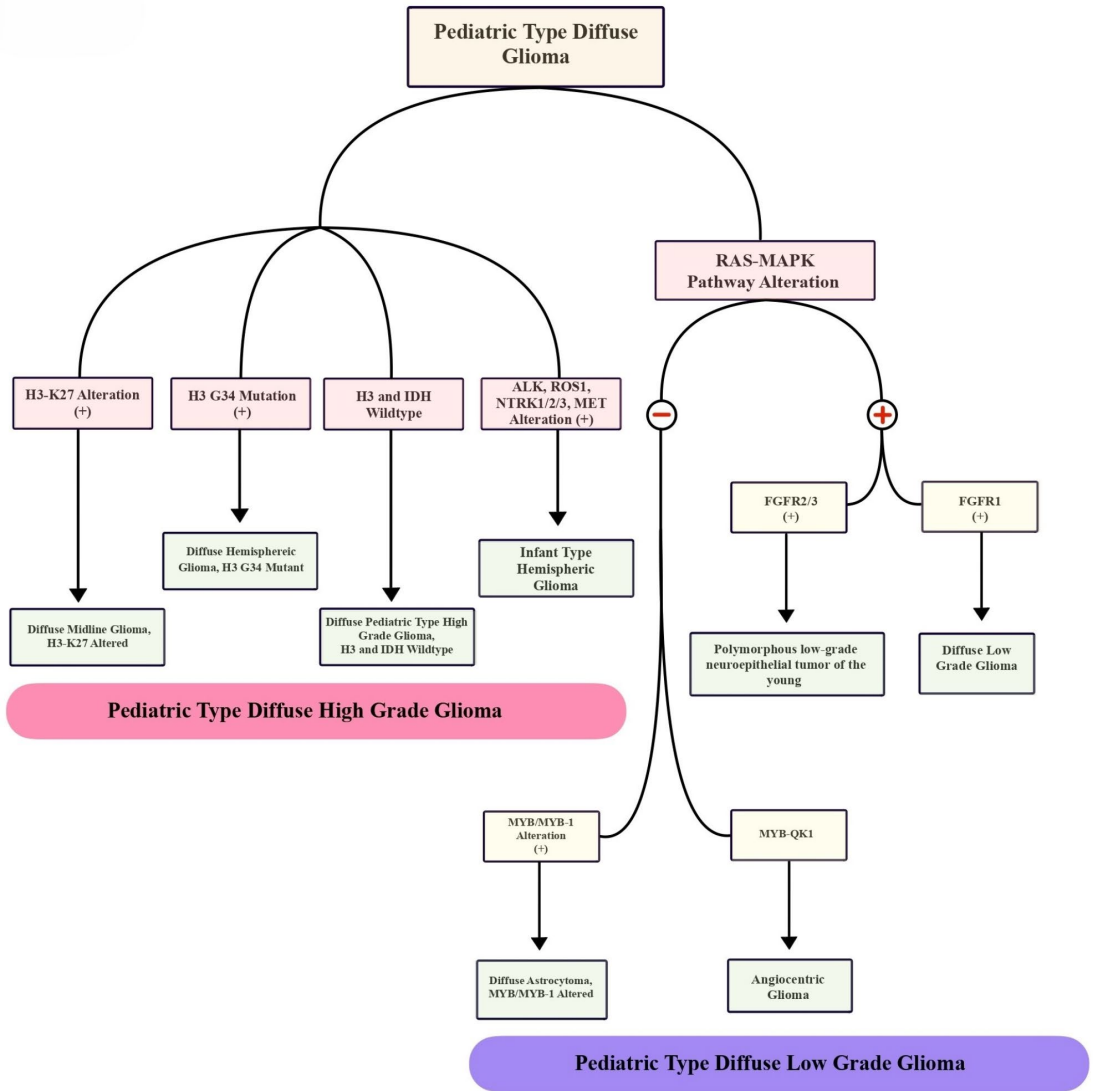
Pediatric type diffuse Glioma classification

Pediatric-type diffuse high-grade gliomas are a separate category of brain tumors that are primarily characterized by their molecular profile. (Fig. 3) The group is further subdivided into various categories, namely diffuse midline glioma with H3 K27 alterations (note that the term “mutant” has been revised to altered), diffuse hemispheric glioma with H3 G34 mutations, diffuse pediatric-type high-grade Glioma with wildtype H3 and IDH status (which encompasses tumors with diverse genotypes), and infant-type hemispheric glioma. Tumors with H3 K27 alteration are classified as WHO Grade-4 and are invariably associated with poor prognosis [29]. Kandemirli et al. [30] evaluated the efficacy of radiomics features in predicting H3 K27 alteration status in midline gliomas, with more than 50% of the tumors studied from pediatric cases. The study found that an XGBoost model was able to achieve an AUC of 0.791 in the training set and 0.737 in the test set, respectively. The presence of the H3K27M mutation in midline gliomas holds significant prognostic implications and represents a potential target for immunotherapy. A radiomics-driven approach using standard MRI sequences holds the potential for accurately predicting the H3K27M mutation status in midline gliomas.

#### H3 G34-mutant

The H3 G34 mutation is a genetic alteration that affects the H3 histone protein, a key regulator of gene expression. In the 2021 CNS-5 WHO classification update, H3 G34 mutation has been recognized as a new molecular subtype of pediatric-type high-grade diffuse Gliomas. These tumors are now designated as “diffuse hemispheric glioma with H3 G34 mutation” and are classified as WHO Grade 4. Patients with these tumors often exhibit poor response to treatment and an unfavourable prognosis. There is a significant paucity of studies describing the radiomics and radiogenomics of H3 G34 mutant tumors. Shao et al. [31] reported that FeAture Explorer (FAE) generated models (AUC = 0.925), based on radiomics features of conventional MR images, demonstrated superior discriminatory ability between H3 G34-mutant and IDH-mutant gliomas compared to Visually Accessible Rembrandt Image (VASARI) feature analysis (AUC = 0.843). According to Lasocki et al. [32], H3 G34 mutant tumors can exhibit a range of marginal and location characteristics, ranging from well-defined to ill-defined. These tumors may demonstrate absent, faint, or mild enhancement, and various enhancements have been reported across multiple studies. Additionally, intra-tumoral calcification, hemorrhage, or cystic changes may be observed, as noted by Vettermann et al. [33] and Kurokawa et al. [34]. Hyper-perfusion may also be seen on Arterial Spin Labelling (ASL), as reported by Puntonet et al. [35]. FET-PET imaging features of eight H3 G34-mutant Gliomas revealed high uptake in all cases, according to Vettermann et al. [33]. The available imaging data on H3 G34 mutant Gliomas are sparse and varied in nature, although some recurring patterns have been identified. Leveraging radiomics-based AI techniques could offer a promising solution to address the inherent heterogeneity within the existing dataset. Other relevant literature has been summarised in Table-1.

**Table 1:** Diagnostic markers

Article	No. of patients	Sequence Used	Segmentation	Software	Type of analysis	Top discriminating feature	Methodology		Results
**IDH Mutation**									
Kasap et al. 2024 [15]	106	T1-w, CET1-w T2-w and FLAIR sequence	Semi-automatic	^a^ 3D slicer	A total of 107 radiomic features were extracted.	age_at_diagnosis, original_shape_Flatness	Radiomics		Results show that CE-T1W images are most optimal to predict IDH mutation status
				^b^ PyRadiomics		original_gldm_LargeDependence, HighGrayLevelEmphasis			
Hosseini et al. 2023 [8]	57	T1- MPRAGE, Axial T2 FLAIR, CE T2W	Manual	^b^ PyRadiomics	A total of 105 original radiomic features from categories (shape, first-order statistical, second-order texture, and higher-order statistic) were extracted	-	Radiomics		Best discriminatory performance (AUC = 0.93, ACC = 0.92) obtained from solid/contrast enhancing, and core tumor overlaid on post-contrast T1-weighted images
							Machine learning		
							Deep Learning		
Liu et al. 2023 [80]	205	T1C, T2, T1 FLAIR, and T2 FLAIR	Manual	^c^ ITK-SNAP	A total of 428 radiomic features (107 from each sequence) were extracted.	GLRLM and contrast features	Radiomics		The LR classifier achieved the best results in predicting the IDH mutation status with an AUC of 0.8572
Calabrese et al. 2022 [6]	199	T1 pre, T1 post, T2, T2/FLAIR, SWI, DWI, ASL, MD, AD, RD, and FA	Automated deep learning-based tumor segmentation followed by Manual correction	^b^ PyRadiomics	Default set of shape features (*n* = 26), first-order grayscale features (*n* = 19), and higher-order grayscale features (*n* = 75) were extracted yielding 5300 radiomics features per patient	-	Radiomics, Deep Learning		AUC/Sens/Spec of 0.96/1.00/0.83 for predicting IDH1 mutation status
				^c^ ITK-SNAP v3.8.0					
				^d^ TensorFlow 2.4					
He et al. 2022 [16]	108 (From TCGA-LGG dataset)	T1, T2, FLAIR, and T1Gd	Manual	^e^ FeAture Explorer (FAE, Version 0.5.2)	“6-Step” general radiomics model to noninvasively predict the IDH mutation status by simultaneously tuning combined multi-sequence MRI proposed	-	Radiomics		Optimal radiomics pipeline for predicting IDH mutation status was the T2 + FLAIR combined multi-sequence MRI with an AUC of 0.873 ± 0.05
Kawaguchi et al. 2021 [81]	TCIA dataset (n = 159), NCCH Japan (n = 166)	T1, T1-CE, T2, FLAIR	Manual	^b^ PyRadiomics	A total of 16,221 features generated for each patient, using both images and clinical records. Inception-ResNet v2 architecture used	-	Deep learning and Machine learning		AUROC of 0.867 achieved for IDH prediction on TCIA dataset, but decreased to 0.383/0.392 in the validation/test NCC cohort
S. Kihira et al. 2021 [82]	111 (Training n = 91, test n = 20)	T2, FLAIR, T1CE, DWI	Manual 2D	^f^ Olea Sphere software	Total of 92 radiomics features assessed	Conventional: FLAIR – GLCM Informal Measurement Correlation 2. T1c+ - First order skewness, GLCM Difference variance, GLSZM Small Area High Gray, GLCM Dependence Variance	Machine learning		Predictive model for IDH1 status based on conventional MR imaging achieved area under the curve (AUC) of 0.95. Upon incorporating diffusion data, combination of 5 conventional and 5 diffusion MR features remained as significant contributors, resulting in a perfect AUC of 1.0.
						Diffusion: B1000: First Order Skewness			
						ADC: First Order Skewness, GLRLM Run Length Non-Uniformity, GLSZMGL Non-Uniformity Norm, GLSZM Small Area High Gray			
Kim et al. 2020 [83]	155	T2W, FLAIR, T1W, DWI, CET1WI, and DSC. DWI	Manual	^g^ MITK software	A total of 6472 radiomic features extracted and analysed	-	Radiomics		Multipara metric MR radiomics achieved an AUC of 0.795/0.747 on the training/validation sets in predicting IDH mutation.
				^h^ Matlab					
Niu et al. 2020 [84]	182	T1CE	Manual 2D	^i^ AK software (Analysis Kit, GE Healthcare)	396 features extracted including histogram & texture (GLCM, RLM, GLSZM) parameters, and form factor parameters.	Volume CC	Radiomics		The model exhibited good discriminatory performance in both the primary dataset (AUC = 0.87, ACC = 0.798, sensitivity = 85.5%, specificity = 75.4%) and the validation dataset (AUC = 0.86, ACC = 0.789, sensitivity = 91.3%, specificity = 69.0%).
						Intensity variability	Machine learning		
						Short run emphasis_angle 90_offset 4			
Park CJ et al. 2020 [85]	168	DTI, T1CE, T2, FLAIR	Manual 2D	^j^ Medical Image Processing, Analysis, and Visualization software package version 7.0	A total of 158 and 253 radiomic features were extracted from DTI and conventional MRI respectively.	-	Radiomics		The combined model incorporating DTI and conventional radiomics demonstrated significantly superior performance compared to the model comprising only DTI histogram parameters and conventional radiomics (AUC: 0.900 vs 0.869, p = 0.040).
				^b^ PyRadiomics			Machine learning		
Peng et al. 2020 [86]	105 (Training n = 73, test n = 32)	T1CE, T2, ASL	Manual 2D	^b^ PyRadiomics	A total of 851 radiomics features extracted from each VOI	-	Radiomics		The classifier, which integrated features from all three sequences, achieved an accuracy of 0.823 and an AUC of 0.770 (P < 0.05).
							Machine learning		
Sakai et al. 2020 [87]	100 (Training n = 60, validation n = 20, test n = 20)	DWI, FLAIR	Manual 2D	^f^ Olea Sphere software	A total of 184 radiomic features extracted. The DWI model utilized 71 out of the 92 features, while FLAIR model utilized 33 out of the initial 92 features.	-	Radiomics		The best performance achieved was with an AUC of 95%, Accuracy of 90%, Precision/Recall/f1-score of 94%/94%/94% for IDH1 wildtype, and 75%/75%/75% for IDH1 mutants
							Machine learning		
Han et al. 2020 [88]	59	Conventional MRI, contrast-enhanced MRI, and APTW imaging	Manual	^c^ ITK-SNAP, Non-commercial Analysis-Kit software (GE Healthcare, China)	A total of 1038 features including 8 first-order histograms, 6 GLCM and 4 GLRLM extracted and analyzed	Run Length Nonuniformity angle0 offset1	Machine learning		SVM model achieved an AUC of 0.952 and 0.84 in the training set and test set, respectively. Efficacy achieved by SVM model superior to that of univariate analysis.
						Correlation All Direction offset4 SD			
Fukuma et al. 2019 [89]	164	T1W, T2W, FLAIR, and GdT1W	Manual	^h^ MATLAB-based image analysis software	Combination of CNN (AlexNet) and Conventional radiomics based approach used for analysis	-	Deep learning		Using the combination of conventional radiomic features and/or patient age, CNN an accuracy of 73.1% achieved in predicting IDH status.
Ren et al. 2019 [90]	57	3D-ASL, T2, T2 FLAIR, DWI	Manual	Custom developed software in Matlab	265 high-throughput radiomic features were extracted on each tumor volume of interest	eADC: short run emphasis (GLRLM), energy (GLGCM), long-run emphasis (GLRLM), energy (GLCM)	Radiomics		The accuracies/AUCs/sensitivity/specificity/PPV/NPV of predicting IDH1(+) in LGG were 94.74%/0.931/100%/85.71%/92.31%/100%
				^k^ Advantage Workstation 4.6, GE Medical Systems			Machine learning		
Wu et al. 2019 [91]	126	T1, T1CE, T2, T2 FLAIR	Manual 2D	^l^ R Software	A total of 704 radiomic features extracted	-	Radiomics		Random Forest (RF) exhibited excellent predictive performance, with accuracy of 0.885 ± 0.041 and AUC of 0.931 ± 0.036. In contrast, neural network (NN) (accuracy 0.829 ± 0.064, AUC 0.878 ± 0.052) and flexible discriminant analysis (FDA) (accuracy 0.851 ± 0.049, AUC 0.875 ± 0.057) displayed comparatively lower predictive performance.
							Machine learning		
Arita et al. 2018 [92]	199	Conventional MRI, ADC, normalized blood volume	Manual	In-house-developed image analyzing software (Developed in Matlab)	A total of 109 radiomic features quantified and collected	Frontal lobe tumor involvement (MNI_str_loc.04) for IDH Mutant	Machine learning		IDH mutation predicted with an accuracy of 0.85 to 0.87, which improved by implementing lesion location information.
				^m^ JMP Pro ver.13		Magnitude of contrast enhancement (Gdzscore_ara.of.Gd.) for IDH wildtype			
Chang et al. 2018 [93]	496 (Divided in 3 cohorts)	MRI: T1, T1-CE, T2, FLAIR	Manual	^n^ Matrix User v2.2	-	-	Deep learning		IDH prediction accuracies of 82.8% (AUC = 0.90), 83.0% (AUC = 0.93), and 85.7% (AUC = 0.94) achieved on the training, validation, and testing sets, respectively.
				^a^ 3D Slicer software (v4.6)					
Liang et al. 2018 [94]	167	MRI: T1,T1-CE,T2, FLAIR	Manual	^o^ MXNet (version 1.0, Apache Software Foundation)	Multimodal Three-Dimensional DenseNet constructed for the analysis	-	Deep learning		Accuracy of 84.6% achieved on the validation dataset. To evaluate the generalizability, transfer learning techniques applied to predict WHO grade status, yielding a high accuracy of 91.4%on validation dataset.
Li et al. 2017 [95]	151	MRI (T1c, T2 FLAIR)	Auto segmentation Manual	^p^ Brainsuite	A total of 671 image features extracted and replaced with 16, 384 CNN features	-	Deep learning based Radiomics		The radiomics method achieved an area under the operating characteristic curve (AUC) of 86% for estimating IDH1, while the AUC for DLR was higher at 92%. Integrating multiple-modality MR images and utilizing DLR further improved the AUC for IDH1 estimation to 95%.
Yu et al. 2017 [96]	110	T2 FLAIR	Auto Segmentation (Using CNN)		Total of 671 high-throughput features were extracted and quantized.	Shape; Sphericity	Radiomics		In the Leave-One-Out Cross Validation (LOOCV) analysis, IDH1 status achieved an estimation accuracy of 0.80, with a sensitivity of 0.83 and specificity of 0.74. AUC reached 0.86, indicating the promising discriminatory capability of the approach in accurately predicting IDH1 status.
						Texture: Large zone high grey-level emphasis	Machine learning		
						Wavelet feature: small zone high gray-level emphasis	Deep learning		
**TERT promoter**									
Chen et al. 2023 [97]	143	Axial T2W, DWI, and ADC	Manual	^a^ 3D slicer software	A total of 2553 features extracted	ADC entropy		Radiomics	Model constructed based on the RFE and LDA achieved the best diagnostic performance (AUC, accuracy, sensitivity, and specificity: 0.964, 0.940, 0.891, and 0.982, respectively) in predicting TERT p mutation
Huo et al. 2023 [98]	109	Axial T2W, CE-T1W, T1W and CE-T1W	Manual	^q^ MRIcron	A Total of 2608 radiomic features extracted for each patient	Wavelet-HHH_glcm_Idmn wavelet-HHH_glcm_Idn exponential_glszm_GrayLevelNonUniformityNormalized wavelet-HLL_glszm_LowGrayLevlZoneEmphasis.		Radiomics	Fusion radiomic model with 4 radiomic features achieved an AUC value of 0.876 and 0.845 in the training and validation set respectively for predicting TERT p mutation in IDH wildtype gliomas.
				^b^ Pyradiomics					
Wang et al. 2023 [99]	140 (With independent validation on 34 separate cases)	T1W, T2W, T1CE, FLAIR, and ADC maps	Auto segmentation and Manual correction	^r^ Niftynet	A total of 3654 radiomic features extracted and analysed	FLAIR_wavelet-LLL_gldm_DependenceVariance		Radiomics	AUROC/sensitivity/specificity 0.952/0.714 /0.963 achieved in the independent validation set in identifying IDHmut pTERTmut tumors
				^c^ ITK-snap		ADC_log-sigma-5-0-mm-3D_firstorder_RootMeanSquared			
				^b^ Pyradiomics version 2.2.0		Visul feature: IDHmut pTERTmut gliomas showed homogenous low-complexity texture			
Zhang et al. 2023 [19]	274 (training n = 156, validation n = 118)	T1CE, T1WI, T2WI	-	-	-	-		Deep learning	DLR signature showed the best discriminative power for predicting TERT promoter mutations, yielding an AUC of 0.990 and 0.890 in the training and external validation cohorts.
Calabrese et al. 2022 [6]	199	T1 pre, T1 post, T2, T2/FLAIR, SWI, DWI, ASL, MD, AD, RD, and FA	Automated deep learning-based tumor segmentation followed by Manual correction	^b^ Pyradiomics ver2.2	Default set of shape features (*n* = 26), first-order grayscale features (*n* = 19), and higher-order grayscale features (*n* = 75) were extracted yielding 5300 radiomics features per patient	-		Radiomics, Deep Learning	AUC/Sens/Spec of 0.75/0.71/0.68 for predicting TERT p mutation status
				^c^ ITK-SNAP v3.8.0					
				^d^ TensorFlow 2.4					
Lu et al., 2022 [100]	176 (training n = 123, validation n = 53)	CE-MRI	-	-	A total of 851 radiomic features extracted.	-		Radiomics	AUC = 0.873 (Validation set) achieved for predicting TERT p mutation
Fang et al. 2021 [18]	164	T2W, CE-T1W	Manual	^h^ Matlab	A total of 1,293 radiomics features from multi-parametric magnetic resonance extracted and analysed	CE-T1WI_Cluster Tendency		Machine learning	An overall accuracy of 0.7988 achieved in predicting TERT P mutation,
						T1WI_Contrast			
						T1WI_Long Run Low Gray Level Emphasis_1			
						T1WI_Low Gray Level Run Emphasis			
						T2WI_Long Run High Gray Level Emphasis_1			
Z. Li et al., 2021 [101]	159	Dynamic [18F] FET PET	Manual	^s^ PMOD view tool	107 radiomic features including first-order statistics, shape-based features, and texture features.	The TTP (Time to peak) model showed the strongest predictive power		Radiomics	0.921/NA/0.82
				^b^ PyRadiomics (v 3.0.1)				Machine learning	
Yan et al. 2021 [102]	357 (training, n = 238 and validation, n = 119)	T1WI, cT1WI, T2WI, T2-FLAIR, and DWI	Manual	^c^ ITK-SNAP	A total of 8730 and 4365 radiomic features extracted for gliomas with peritumoral edema, and without edema respectively.	Tumor_log-sigma-5-0-mm-3D_firstorder_10Percentile		Radiomics	Image fusion model integrating radiomic signatures from contrast-enhanced cT1WI and ADC achieved an AUC of 0.884 and 0.669 for predicting IDH and TERT status, respectively.
				^b^ Pyradiomics 2.0.0		Tumor_wavelet-LL_firstorder_Skewness			
						Tumor_gradient_glszm_LargeAreaHighGrayLevelEmphasis			
						Tumor_original_shape_Sphericity			
						Tumor_log-sigma-1-0-mm-3D_firstorder_Median			
Jiang et al. 2020 [103]	116 (training n = 83, validation n = 33)	CE-T1W, T2W	Manual	^c^ ITK-SNAP	A total of 107 radiomic features extracted, including 14 shape features, 18 first order features, and 75 texture features for each ROI at each modality	Correlation		Radiomics	The tumoral signature model yielded the best performance, with area under the ROC curves (AUC) of 0.948 the training cohort and 0.827 in the validation cohort.
				^b^ PyRadiomics (2.1.0)		Gray-level non-uniformity normalized			
				^t^ Scikitlearn (v0.20.0)		Large dependence high gray-level emphasis			
						Large dependence low gray-level emphasis			
Fukuma et al. 2019 [89]	164	T1W, T2W, FLAIR, and GdT1W	Manual	MATLAB-based image analysis software	Combination of CNN (AlexNet) and Conventional radiomics based approach used for analysis	-		Deep learning	CNN features succeeded in capturing characteristics of TERT p mutation, not identified by conventional radiomic features and patient age. Accuracy of 84.0% achieved using CNN features.
**EGFR amplification**									
Calabrese et al. 2022 [6]	199	T1 pre, T1 post, T2, T2/FLAIR, SWI, DWI, ASL, MD, AD, RD, and FA	Automated deep learning-based tumor segmentation	^b^ Pyradiomics ver2.2	Shape features (n = 26), first order grayscale features (n = 19), and higher order grayscale features (n = 75).	-		Radiomics, Deep Learning	AUC/Sens/Spec of 0.70/0.66/0.68
S. Kihira et al. 2021 [82]	111 (Training n = 91, test n = 20)	T2, FLAIR, T1CE, DWI	Manual 2D	^h^ Matlab	Total of 92 radiomics features assessed	FLAIR – First order skewness		Machine learning	AUC/Sensitivity/Specificity: 0.65/0.68/0.83
						FLAIR – GLSZM Small area emphasis			
						T1c + GLDM Small dependence low gray			
Pasquini L. et al. 2021 [21]	156	MPRAGE, T1w, T2w, T2-FLAIR, DWI, DSC MRI	Manual 2D	^h^ Matlab	Radiomic set included 14 shape features, 18 intensity features, and 75 texture features	rCBV		Machine learning	Accuracy 81%; ROC 74.3%.
B. Sohn et al. 2021 [22]	418 (Training n = 292, test n = 126)	CE-T1w, T1w, T2w, T2-FLAIR	Auto-Segmentation	Python 3 with ScikitLearn library v0.21.2 and the R software	A total of 660 radiomic features were extracted	Run entropy (T1WI, CE mask), high gray-level cone emphasis (T2WI, CE mask), and inverse variance (T1WI, T2 mask).		ML (Binary relevance and Ensemble classifier chain)	AUC/Sensitivity/Specificity: 0.812/0.585/0.743
Li et al. 2018 [104]	270 (Training n = 200, test n = 70)	T2w	Manual 2D	^h^ Matlab	431 texture features (Divided in 4 groups, first order statistics, shape and size features, texture features, wavelet features)	Radiomic signature, comprising 25 first-order statistics or related wavelet features, one shape- and size-based feature, and 15 textural features or related wavelet features		Radiomics, Machine learning	41 MRI features achieved accuracies of 82.5% (AUC = 0.90) in the training set (n = 200) and 90.0% (AUC = 0.95) in the validation set (n = 70)
Hu LS et al. 2017 [105]	25 (GBM)	T1CE, DTI, DSC, PWI	Manual 2D	^c^ ITK SNAP	A total of 336 features extracted composed of 56 features across 6 MR contrasts.	T2.Information.Measure.of.Correlation.2_Avg_1		Radiogenomics, Machine learning	On validation set model achieved 78% accuracy amongst the sample predictions with lowest uncertainty
						T2.Angular.Second.Moment_Avg_1			
						T2.Kurtosis			
						rCBV.Contrast_Avg_1			
Kickingereder et al. 2016 [106]	152	MP-RAGE, T2FLAIR	Manual	^h^ Matlab	A total of 31 features extracted including mutiparametric and multiregional information with histogram quantification of tumor volumes, volume ratios, apparent diffusion coefficients, cerebral blood flow, cerebral blood volume, and intratumoral susceptibility signals.	Increased Gaussian-normalized relative cerebral blood volume and Gaussian-normalized relative cerebral blood flow values		Radiomics, Machine learning	Accuracy of 63% achieved in predicting EGFR amplification status using ML.
**+ 7/-10 copy number change**									
Calabrese et al. 2022 [6]	199	T1 pre, T1 post, T2, T2/FLAIR, SWI, DWI, ASL, MD, AD, RD, and FA	Automated deep learning-based tumor segmentation followed by Manual correction	^b^ Pyradiomics ver2.2	Default set of shape features (*n* = 26), first-order grayscale features (*n* = 19), and higher-order grayscale features (*n* = 75) were extracted yielding 5300 radiomics features per patient	-		Radiomics, Deep Learning	AUC/Sens/Spec of 0.79/0.69/0.76 for predicting + 7/-10 copy number change
				^c^ ITK-SNAP v3.8.0					
				^d^ TensorFlow 2.4					
**1p/19q Co-deletion**									
Kihira et al. 2023 [28]	103	T2-FLAIR	Manual	^u^ Oleasphere	92 radiomic texture features extracted from each VOI in a patient	Radiomics model outperformed the T2-FLAIR sign		Radiomics, Machine learning	AUC/Sensitivity/Specificity/Accuracy: 0.80/87.5%/89.9%/88.8%.
Casale et al. 2021 [107]	209 (training n = 159, validation n = 50)	T2 FLAIR, T1CEN	Manual	^v^ Weka software version 3.8.3	5352 radiomics features per patient extracted from both T1- and T2- weighted images. After correlation-based feature subset selection, 48 features remained for cubic interpolation and 51 features for linear interpolation.	-		Radiomics, Machine learning	Cubic interpolation (AUC/Sensitivity/Specificity/Accuracy: 0.81/0.77/0.85/0.86)
				^h^ Python 3.7.6 version					
				^w^ MIM software version 6.9.0					Linear interpolation (AUC/Sensitivity/Specificity/Accuracy: 0.76/0.72/0.81/0.82)
Yan et al. 2021 [102]	357 (training, n = 238 and validation, n = 119)	T1WI, cT1WI, T2WI, T2-FLAIR, and DWI	Manual	^c^ ITK-SNAP	A total of 8730 and 4365 radiomic features extracted for gliomas with peritumoral edema, and without edema respectively.	Edema_log-sigma-5-0-mm-3D_firstorder_RootMeanSquared		Radiomics	CT1WI based radiomic signature yielded an AUC value of 0.815 in predicting 1p/19q status.
				^b^ Pyradiomics 2.0.0		Tumor_log-sigma-1-0-mm-3D_firstorder_Mean			
						Edema_gradient_firstorder_Kurtosis			
						Tumor_log-sigma-3-0-mm-3D_glcm_ClusterShade			
						Tumor_log-sigma-5-0-mm-3D_firstorder_90Percentile			
Kong et al. 2020 [108]	96 (training n = 78, validation n = 18)	T1CE, T2	Manual	^c^ ITK-SNAP	A total of 107 radiomics features were extracted from the region of interest (ROI) of each imaging modality using PyRadiomics	Informational Measure of Correlation 2		Radiomics, Machine learning	The 3D-radiomics signature displayed an accuracy of 0.897 and AUC of 0.940 in the training dataset, and an accuracy of 0.833 and AUC of 0.889 in the validation dataset.
						Correlation			
				^b^ Pyradiomics		Dependence Entropy			
						Major Axis Length			
Kocak et al. 2020 [109]	107	CET1W, T2W	Semi-automatic	^x^ LIFEx	A total of 84 radiomic features extracted	HISTO-Skewness (T2W)		Radiomics, Machine learning	AUC and accuracy values of five tested algorithms ranged from 0.769 to 0.869 and from 80.1 to 84%, respectively. Neural network had the highest mean rank with mean AUC and accuracy values of 0.869 and 83.8%, respectively.
						GLZLM-HGZE (T2W)			
						HISTO-Entropy-log10 (T2W)			
Batchala et al. 2019 [13]	102 (Data obtained from TCIA)		Manual	-	-	texture		-	Accuracy = 86.3% achieved for predicting the 1p/19q codeletion status
						T2* susceptibility blooming			
						T2-FLAIR mismatch sign			
						Location			
						Midline shift			
Van der Voort et al. 2019 [110]	284 (With validation on 129 cases from TCIA)	T1W, T2W, T2FLAIR	Manual	^c^ ITK-SNAP	A total of 78 image features (such as image intensity, tumor texture, tumor shape, and tumor location) extracted Age and sex added to yield as total of 80.	Cranial/caudal location of the tumor, skewness of the T2-weighted SI histogram, and one of the texture features, together with age and sex		Radiomics, Machine learning	An AUC of 0.72 yielded in the external validation dataset. Higher predictive performance than the average of the neurosurgeons (AUC 0.52) but lower than that of the neuroradiologists (AUC of 0.81)
Han et al. 2018 [111]	277 (training n = 184, validation n = 93)	T2	Manual	^c^ ITK-SNAP	647 radiomic features consisting of shape and size features (8), first order statistics features (17), textural features (54) were extracted for the original image set, and first order statistics and textural features for 8 wavelet filtered image sets.	ori_ fos_skewness (degree of distortion for the image)		Radiomics, Machine learning	The radiomics signature displayed good performance on both the training and validation cohorts with areas under the curve (AUCs) of 0.887 and 0.760, respectively. Results outperformed the clinical model, which demonstrated AUCs of 0.580 and 0.627 on the training and validation cohorts, respectively.
						Coif5_glcm_covariance (measurement of heterogeneity in an image filtered with low-pass in the x-direction and high-pass in the y- and z-direction)			
						Coif2_glcm_ sum_variance (the change frequency and period of the texture in an image filtered with low-pass in the x- and y-direction and high-pass in the z-direction)			
Lu et al. 2018 [112]	214 (with independent validation on a set of 70 patients)	T1W, CET1W, T2W, FLAIR, DWI	Semi-automatic with Manual correction	Home-made software, MR Radiomics Platform (MRP) Graphic interface built in Matlab	A maximum of 39,212 MR radiomic features generated for each subject.	Texture measurements describing spatial variations of tumor intensity found to be the most illustrative for the IDH and 1p/19q genotypes		Radiomics, Machine learning	An AUC of 0.922 achieved on the training dataset while and Accuracy of 80% yielded in predicting the 1p/19q co deletion status.
Akkus et al. 2017 [113]	159 Low grade gliomas patients	T2 and post-contrast T1-weighted MR	Semi-automatic	Semi-automatic LGG segmentation software (^p^ STAPLE software)	-	-		CNN (Deep learning)	Best performing configuration of CNN-architecture outperformed the classical ML model (SVM) with 93.3% (sensitivity), 82.22% (specificity), and 87.7% (accuracy)
Shofty et al. 2017 [114]	47	T2W, T1W, FLAIR	-	-	A total of 152 features, including size, location and texture, extracted.	-		Radiomics, Machine learning	Ensemble Bagged Trees classifier achieved best results with an AUC/Sens/Spec of 0.87/92/83 in predicting 1p/19q co deletion status.
**H3 K27**									
Kun et al. 2023 [115]	103	CE-T1W	Manual	^g^ Medical Imaging Interaction Toolkit (MITK)	A total of 1781 radiomic features extracted and combined with conventional MR and clinical features to construct an integrated model	Age, 2 radiomics features, and 3 conventional MRI features were the 6 most significant features		Radiomics, Machine learning	Integrated model (Radiomic + Clinical features) achieved an optimal AUC/accuracy of 0.98/0.903 in the testing cohort.
				^b^ Pyradiomics (version 3.0.1)					
Yang et al. 2023 [116]	126	T1W, dMRI		-	21 radiomics features and 52 topological properties of brain structural connectivity network selected to construct a machine learning-based H3K27M mutation prediction model	-		Radiomics, Machine learning, Connectomics	AUC/Accuracy of 0.9246/92.11% achieved in predicting H3 K27 alteration. Combined multivariate logistic model built using T1 and dMRI based signature achieved an AUC of 0.8783 in the validation cohort
Li et al. 2023 [117]	418 (Diffused Midline Glioma patients) 133 (Spinal Cord Glioma Patients)	T2W	-	-	-	-		Deep Learning	Predictive accuracies, sensitivities, and specificities of H3 K27M mutation status were 92.1%, 98.2%, 82.9% in the testing cohort (In Diffuse Midline Glioma patients).
Guo et al. 2022 [54]	102 (training n = 72, test n = 30)	T2WI, T1WI, FLAIR, CE-T1WI, SWI, DWI, and DSC-PWI	Manual	^c^ ITK SNAP	Set of 851 features was extracted from each sequence image	-		Radiomics, Machine learning	The radiomics models based on multiparametric MRI demonstrated high accuracy in predicting the H3 K27M mutant status in diffuse midline gliomas (DMG), with AUC values ranging from 0.807 to 0.969 for different sequences or sequence combinations. The study identified the optimal model as one that utilized a combination of all sequences, achieving an AUC of 0.969.
				^e^ FeAture Explore (V 0.4.2) using PyRadiomics					
				^l^ R software 3.5.3					
Wu et al. 2022 [55]	107	CE-T1W, FLAIR, DWI, ADC	Manual	^b^ Pyradiomics	A total of 4520 radiomics features were extracted. Nine radiomics features selected among all extracted features to construct the radiomics signature	-		Radiomics, Machine learning	Ring enhancement was found to be a significant and independent clinical predictor (p < 0.01). Constructed nomogram, incorporating radiomics signature and ring enhancement, yielded an area under the curve (AUC) values of 0.95 and 0.90 in the training and testing sets, respectively.
				^c^ ITK-SNAP					
Kandermirli et al. 2021 [30]	109	Nonenhanced T1-W, T2-W, T2-FLAIR, postcontrast T1-W, Apparent diffusion coefficient (ADC) maps	Manual	^y^ SimpleITK	A total of 651 radiomic features per each sequence extracted	LoG-sigma 2 mm three dimensional first-order maximum on ADC maps		Radiomics, Machine learning	The study evaluated two models, and the XGBoost model with additional feature selection yielded superior results. The AUC for this model was 0.791 and 0.737 in the training and test set respectively.
				^b^ PyRadiomics		Wavelet-LH first-order range on T2-WI			
						Wavelet-LH gray-level dependence matrix large dependence high gray-level emphasis on ADC maps			
						Original first-order mean absolute deviation on ADC maps			
						LoGsigma 4 mm three-dimensional first-order maximum on FLAIR			
Li et al. 2021 [118]	30	T1WI, T2WI, CE-T1WI	Manual	^c^ ITK SNAP	272 radiomic features were extracted from MR images of each tumor, grouped into seven categories: shape features, first-order features, GLCM features, GLRLM features, GLSZM features, and GLDM features.	T2WI		Radiomics, Machine learning?	Cyst formation exhibited a significant difference between DGM-M and DGM-W tumors (p = 0.024) among the visually accessible features. However, there were no significant differences observed between DGM-M and DGM-W tumors for necrosis (p = 0.191) hemorrhage (p = 0.657), and the T1/T2 ratio (p = 0.689).
				^b^ PyRadiomics					
Zhuo et al. 2021 [119]	81 (training n = 64, test n = 17)	T1W, T2W, T2-FLAIR, DWI, CE-T1W, APTw	Manual	^a^ 3D Slicer	A total of 1316 radiomic features were obtained from 3D tumor masks.	-		Radiomics, Machine learning	Utilizing support vector machine (SVM) to identify radiomic features derived from amide proton transfer-weighted (APTw) imaging, a high accuracy rate of 0.99 (63/64) was achieved for the retrospective cohort's prediction of H3K27M-mutant tumors in the training set. In the test set, the accuracy was 0.88 (15/17).
				^e^ Feature Analysis Explorer v0.3.6					
Su et al. 2020 [120]	100 (training n = 75, testing n = 25)	T1W, CE-T1W, T2W, T2 FLAIR	Manual	^c^ ITK SNAP	A total of 18 first-order features, 13 shape features, 22 GLCM features, 16 GLRLM) features, and 16 GLSZM features were extracted	original_glszm_GrayLevelVariance original_firstorder_10percentile		Radiomics, Machine learning	Out of the 10 models evaluated, the highest-performing one achieved an AUC of 0.903 in the training cohort and 0.85 in the validation set.
				^b^ PyRadiomics		original_shape_Maximum2DDiameterSlice			
				^l^ R package ver 3.6		original_shape_SurfaceVolumeRatio original_shape_Volume			
Pan et al. 2019 [121]	151	T1W, CE-T1W, T2W, CE-T2W	-	-	A total of 1697 features, including 6 clinical parameters and 1691 imaging features extracted	-		Machine learning	Machine learning-based model achieved an accuracy of 84.44% (AUC of 0.8298) in the test cohort for predicting H3 K27 mutation. The simplified model achieved an AUC of 0.7839 in the test cohort.
Liu et al 2018 [122]	55 (training n = 38, validation n = 4, testing n = 13)	T1w-MPRAGE	Auto segmentation (Deep learning)	^a^ 3D slicer version 4.1	Cascaded two-task framework for segmentation and H3 K27 status prediction using CNN	CNN30 + SVM		Deep learning	The CNN-feature based method outperformed the traditional hand-crafted feature based method by at least 17% and 0.30, with a prediction accuracy (ACC) of 96.52% and an AUC of 0.953
**H3 G34**									
Shao et al. 2024 [123]	53	-	-	^b^ PyRadiomics	Visually Accessible Rembrandt Images (VASARI) features and radiomic features extracted	-		Radiomics	FAE-generated model, based on radiomics features (AUC 0.925), displayed better discriminatory performance between G34m-DHG and IDH-WT-GBM than VASARI feature analysis (AUC 0.843).
								Machine learning	

^a^ https://www.slicer.org/

^b^ https://www.radiomics.io/pyradiomics.html

^c^ http://www.itksnap.org/pmwiki/pmwiki.php

^d^ https://tensorflow.org/

^e^ https://github.com/salan668/FAE

^f^ https://www.olea-medical.com/en/

^g^ https://www.mitk.org/wiki/The_Medical_Imaging_Interaction_Toolkit_(MITK)

^h^ MathWorks, Natick, Massachusetts

^i^ https://www.gehealthcare.com/products/healthcare-it

^j^ https://mipav.cit.nih.gov/

^k^ https://www.gehealthcare.com/education/advantage-workstation-for-diagnostic-imaging

^l^ R statistical and computing software (http://www.r-project.org)

^m^ https://www.jmp.com/en_us/software/predictive-analytics-software.html

^n^ https://www.mathworks.com/matlabcentral/fileexchange/43780-matrixuser-v2-2

^o^ https://mxnet.apache.org/versions/1.9.1/

^p^ https://brainsuite.org/

^q^ https://www.nitrc.org/projects/mricron

^r^ https://niftynet.io/

^s^ https://www.pmod.com/files/download/v31/doc/pbas/877.htm

^t^ https://scikit-learn.org/stable/whats_new/v0.20.html

^u^ https://ml.cms.waikato.ac.nz/weka/

^v^ https://www.mimsoftware.com/cve-2023-30262

^w^ https://www.lifexsoft.org/

^x^ http://crl.med.harvard.edu/research/staple/

^y^ https://simpleitk.org/

## Prognostic markers

### MGMT promoter methylation

MGMT (O6-Methylguanine-DNA Methyltransferase) is a well-established prognostic and diagnostic biomarker for CNS tumors. Methylation of the MGMT promoter region is associated with improved response to alkylating chemotherapy and radiation therapy. For instance, MGMT can counteract the effect of temozolomide via transfer of methyl groups from targeted guanine bases to MGMT [36]; silencing MGMT expression therefore is associated with improved sensitivity to Temozolomide. MGMT promoter methylation has been extensively analysed in radiomic and radiogenomic studies. Korfiatis et al. [37] reported that the best classification system, a Support Vector Machine (SVM) based classifier had an AUC/sensitivity/specificity of 0.85/0.803/0.813 with correlation, energy, entropy, and local intensity as the top differentiating features of MGMT promoter methylation. Kanas et al. [38] predicted the MGMT promoter methylation status with an accuracy/sensitivity/specificity up to 0.736/0.853/0.760 using a wrapper-based approach to select the most informative variables such as edema/necrosis volume ratio, tumor/necrosis volume ratio, edema volume, and tumor location and enhancement characteristics. ADC has also emerged as a promising surrogate biomarker in the detection of MGMT status [39]. Utilizing algorithms such as SVM, along with texture features extraction, offers a viable method to predict MGMT methylation status in CNS tumors. Incorporating additional MR imaging methods, like ADC, holds the potential to enhance the accuracy of this approach even further.

### CDKN2A/B

CDKN2A/B (Cyclin Dependent Kinase Inhibitor) homozygous deletion emerges as an independent prognostic marker in all grades of IDH-mutant astrocytomas, including grade 4 astrocytoma. With recent changes introduced in the new WHO CNS-5 grading, a tumor with CDKN2A/B is designated as WHO Grade 4 irrespective of vascular and necrosis status. Multiple studies have tried to predict the CDKN2A/B alteration in CNS tumors using imaging features with variable degrees of success. Park et al. in their paper [40] showed that infiltrative pattern and 95th percentile normalized rCBV are independent predictors of CDKN2A/B homozygous deletion with an AUC/accuracy/sensitivity/specificity of 0.830/0.904/0.833/0.750. Calabrese et al. [6] used a combined radiomics and CNN architecture to predict CDKN2A/B homozygous deletion status with an AUC/accuracy of 0.86/0.79. Yang et al. [41] was able to predict the CDKN2A/B homozygous deletion status with an AUC of 0.880 and 0.825 across the training and validation sets using a comprehensive radiomic-based and clinical model. Radiomic models exhibits high accuracy and reliability in predicting CDKN2A/B homozygous deletion so they can be employed to further refine diagnosis and facilitate informed clinical decision-making.

**Table 2:** Prognostic markers

Article		No. of patients		Sequence Used		Segmentation			Software/Packages	Type of analysis	Top discriminating feature		Methodology	Results	
**MGMT p methylation**															
Qureshi et al. 2023 [124]		2000 (Publicly available BRATS-2021 dataset)		T1w, T2, FLAIR, and T1Gd mpMRIs.		-			^a^ Cancer Imaging Phenomics Toolkit (CaPTk)	Novel two-stage MGMT Promoter Methylation Prediction system involving extraction of latent features fused with radiomic features predicting the genetic subtype of glioblastoma	-		Deep learning, Machine learning, Radiomics	Highest classification performance achieved an Accuracy/Sensitivity/Specificity of 96.84/96.08/97.44 to predict MGMT methylation status in patients suffering from Glioblastoma.	
									^b^ Federated Tumor Segmentation (FeTS) tool						
Do et al. 2022 [125]		53		T1w, T1-Gd, T2w, and T2-FLAIR		Already Segmented features obtained from a previous study			^c^ Scikit-learn package	Total 704 radiomics features were obtained from a previous study and classified into seven categories	TEXTURE_GLRLM_ED_T2_GLV TEXTURE_GLSZM_NET_T1_SZE HISTO_ET_T2_Bin6		Radiomics, Machine learning	Cross-validation results show GA-based wrapper model yielded an Accuracy/sensitivity/specificity of 0.925 /0.894/0.966 for predicting the MGMT methylation status in GBM	
S. Kihira et al. 2021 [82]		111 (Training n = 91, test n = 20)		T2, FLAIR, T1CE, DWI		Manual 2D			^d^ Matlab	Total of 92 radiomics features assessed	Conventional: FLAIR - First Order Mean Absolute Deviation. T1c+ - GLCM Cluster Shade. Diffusion: B1000 - GLCM-Auto Correlation, GLCM Cluster Shade. ADC - GLCM Sum Entropy		Machine learning	AUC/Sensitivity/Specificity/Accuracy/Threshold: 0.79/70/65/67/0.51	
Wei et al. 2019 [126]		105 (training n = 71, validation n = 31)		T1CE, T2, FLAIR, ADC maps		Manual			^e^ R software	A total of 3,051 imaging features extracted including textural and non-textural features	Oedema degree was the top differentiating feature		Radiomics, Machine learning	The fusion radiomics signature combining the four single radiomics signatures was constructed with a Rad-score. The signature achieved optimal AUC values of 0.925 and 0.902 in the training and validation cohorts, respectively.	
Jiang et al. 2019 [127]		122 (training n = 87, validation n = 35)		T1 (3D-CE-T1), T2		Manual			^f^ ITK-SNAP	1702 radiomics features extracted, consisting of 14 shape, 18 first-order, 75 texture, and 744 wavelet features.	-		Radiomics, Machine learning	The fusion radiomics model, created by combining both series, demonstrated superior performance. In the training dataset, it achieved an accuracy of 0.849 and an area under the curve (AUC) of 0.970. In the validation dataset, the model had an accuracy of 0.886 and an AUC of 0.898.	
									^g^ Pyradiomics						
									^e^ R 3.4.1						
									^h^ Python 3.6.5						
Li et al. 2018 [104]		193 (training n = 133, validation n = 60)		Multiparametric		Auto Segmentation (Using CNN based model)			^i^ TensorFlow	1,705 multiregional radiomics feature extracted and analysed	GLSZM_Small Zone Low Grey Level Emphasis, NGTDM_Business (spatial rate of intensity change within the tumor core)		Radiomics, Machine learning, Deep learning	The radiomics model, achieved an area under the curve (AUC) of 0.88 and an accuracy of 80%, in predicting mgmt. methylation status. Combing clinical factors with radiomics features yielded no benefit to prediction performance	
									^e^ R Software						
Xi et al. 2018 [128]		98 (independent validation done separately on n = 20 patient)		T1, T1CE, T2		Manual			^j^ MITK	A total of 1665 radiomics features extracted, quantized, and reduced using LASSO regularization.	-		Radiomics, Machine learning	The optimal classification system for predicting MGMT promoter methylation status was obtained through the amalgamation of 36 T1 WI, T2 WI, and enhanced T1 WI image features. This system demonstrated a high accuracy rate of 86.59%. Further validation was performed yielding comparable results, with an accuracy of 80%.	
									^d^ Matlab						
Korfiatis et al. 2016 [37]		155		T1WI, T2WI, CE-T1WI		Semi-automatic			^f^ ITK SNAP	-	-		Machine learning	AUC/sensitivity/specificity of 0.85/0.803/0.813 achieved using a SVM based classifier	
**CDKN2A/2B**															
Zhang et al. 2023 [129]		234(Obtained from TCIA and TCGA)		T1WI, T2WI CE-T1WI, T2FLAIR		-		-		Two independent multi-sequence networks (ResFN-Net and FN-Net) were constructed on the basis of ResNet and ConvNeXt network.	-		Deep Learning	ResFN-Net and FN-Net achieved and AUC/Accuracy of 0.8804/0.813 and 0.9704 /0.9236 respectively in predicting CKDN2A/B homozygous deletion status.	
Gao et al. 2023 [130]		251		CE-T1W, T2-FLAIR		-		-		1106 radiomics and 1000 deep learning features extracted. Radiomics models, deep learning-based radiomics models and final integrated model combining radiomics features with deep learning features compared	-		Deep learning, Radiomics	Combined model (training AUC = 0.966; validation AUC = 0.935; test group: AUC = 0.943) outperformed the optimal models based on only radiomics or DLR features (training: AUC = 0.916 and 0.952; validation: AUC = 0.886 and 0.912; test group: AUC = 0.862 and 0.902).	
Yang et al. 2023 [41]		292		GD-T1W, T2 FLAIR		Manual		^g^ PyRadiomics (ver 2.2)		1688 radiomic features extracted, divided into four categories: first order features, shape, texture, higher order statistical features	Comprehensive (Clinical + radiomics) model		Radiomics, Machine Learning	The comprehensive model (clinical + radiomics) achieved AUCs of 0.880 and 0.825 in the training and validation sets, respectively.	
								^h^ Python (version 3.7.0)							
								^c^ Scikit-learn version 0.19.2							
								^e^ R software							
Park et al. 2023 [40]		88		T1W, T2W, FLAIR,3D CE-T1W, DWI and DSC images		Auto Segmentation		^k^ HD-GLIO Segmentation tool		-	Infiltrative pattern, Maximal diameter, nCBV		Conventional radiomics	An infiltrative pattern, larger maximal diameter, and higher 95th percentile of nCBV were independent predictors of CDKN2A/B homozygous deletion status, with an AUC of 0.83.	
Calabrese et al. 2022 [6]		199		T1 pre, T1 post, T2, T2/FLAIR, SWI, DWI, ASL, MD, AD, RD, and FA		Semi-automated deep learning-based tumor segmentation, followed by manual correction		^g^ PyRadiomics ver2.2		A total of 5300 radiomics features were extracted from each patient, including default set of shape features (n = 26), first-order grayscale features (n = 19), and higher-order grayscale features (n = 75).	CNN model outperformed the radiomics model.		Radiomic, Deep Learning	AUC/Sens/Spec of 0.86/0.82/0.73 in predicting CDKN2A/B status using combined Radiomics, CNN approach.	
								^f^ ITK-SNAP v3.8.0							
								^i^ TensorFlow 2.4							
**Medulloblastomas**															
Chang et al. 2021 [42]	38		T1WI, T2WI, FLAIR, CET1, and DWI		Manual		-			A total of 253 radiomic features extracted from each tumor	Features extracted from CET1 GLCM features by using sequential forward selection algorithm delivered the highest performance to differentiate 4 molecular subgroups	Radiomics, Machine learning			The WNT and G3 groups had higher values in Cluster Tendency, Contrast, Difference entropy, and Dissimilarity, while the SHH and G4 groups had higher values in Entropy, Inverse Difference Normalized (IDN), Inverse Difference Moment Normalized (IDMN), and Cluster Prominence, for the 4 and remaining 4 textural features, respectively. All features represent local patterns in tumors.
Iv et al. 2019 [131]	109		2D T1WI, 2D T2WI, 3D T1WI, T1WI, T2WI		Manual		^l^ ePAD			A total of 590 MR imaging–based radiomic features were extracted from the tumor ROIs	Lesion area, edge-sharpness, LAII, and histogram features, with edge-sharpness features being the most important for predicting SHH and group 4.	Radiomics, Machine Learning			Double 10-fold cross-validation model for predicting sonic hedgehog, group 3, and group 4 tumors achieved an AUC = 0.79, 0.70, and 0.83, respectively. With the independent 3-dataset cross-validation strategy, select radiomic features were predictive of sonic hedgehog (AUC = 0.70–0.73) and group 4 (AUC = 0.76–0.80) medulloblastoma.
Yan et al. 2020 [132]	122 (training = 92, testing n = 30)		T1WI, T2WI, FLAIR, DWI, and CE-T1WI		Manual		^g^ PyRadiomics			In total, 5,929 quantitative features were extracted from five MRI sequences for each patient.	Tumor location, Hydrocephalus, Radiomic features (n = 11)	Radiomics, Machine learning			A model using 11 radiomic features achieved high accuracy (AUC of 0.8264) for WNT tumors, and modest AUCs of 0.6683, 0.6004, and 0.6979 for SHH, Group 3, and Group 4 in the testing cohort, respectively. Addition of location and hydrocephalus into the radiomics model resulted in improved AUCs of 0.8403 and 0.8317 for WNT and SHH, respectively. Adding gender and age, the AUCs for WNT and SHH were further improved to 0.9097 and 0.8654, while the accuracies were 70 and 86.67% for Group 3 and Group 4, respectively.
							^e^ R software								

^a^ https://www.med.upenn.edu/cbica/captk/

^b^ https://www.med.upenn.edu/cbica/fets/

^c^ https://scikit-learn.org/stable/

^d^ MathWorks, Natick, Massachusetts

^e^ R statistical and computing software

^f^ http://www.itksnap.org/pmwiki/pmwiki.php

^g^ https://www.radiomics.io/pyradiomics.html

^h^ https://www.python.org/

^i^ https://www.tensorflow.org/tutorials/images/classification

^j^ https://www.mitk.org/wiki/The_Medical_Imaging_Interaction_Toolkit_(MITK)

^k^ https://github.com/CCI-Bonn/HD-GLIO

^l^ https://epad.stanford.edu/

### Medulloblastoma (SHH activated, WNT activated)

In line with 2016 guidelines, the updated CNS-5 classification classifies Medulloblastomas as either molecularly or histologically defined. The molecular subgroups of MBs are determined based on DNA methylation or transcriptome profiling and have remained unchanged. These molecular subgroups include medulloblastoma, WNT-activated (a); medulloblastoma, sonic hedgehog [SHH]-activated and TP53 wild-type (b); medulloblastoma, SHH-activated and TP53-mutant (c); and medulloblastoma, non-WNT/non-SHH (d). Chang et al. [42] conducted a study to compare 253 Radiomic image features across four molecular subtypes of medulloblastoma. The results showed that six features exhibited significant differences between three groups of medulloblastoma, while two features showed significant differences between all four groups. Among the four features demonstrating higher values in the WNT and G3 groups compared to the SHH and G4 groups, namely cluster tendency, contrast, difference entropy, and dissimilarity, all were textural features depicting local patterns in tumors. Conversely, the remaining four features, including entropy, Inverse Difference Normalized (IDN), Inverse Difference Moment Normalized (IDMN), and cluster prominence, showed higher values in the SHH and G4 groups than in the WNT and G3 groups. Distinct radiomic signatures associated with different molecular types of Medulloblastomas can help in quick and easy classification of molecular profiles, allowing for a targeted treatment approach and thereby improving prognosis across all subgroups [43]. Other relevant literature has been summarised in Table-2.

## Discussion

The recent update in the WHO 2021 CNS-5 guidelines has introduced multiple revisions to the classification and grading of CNS tumors. In previous WHO modules, tumor classification was primarily based on microscopic characteristics and tumor histopathology. However, the updated criteria now incorporate genetic information for specific tumor types, leading to the reclassification of certain entities. These changes reflect the acknowledged impact of genetic factors on tumor development and subsequent treatment strategies.

Currently, stereotactic brain biopsy (SBB) is the gold standard for diagnosing and classifying CNS tumor histopathology and related molecular mutations. Yet, the technique has its limitations due to the complexity and heterogeneity of gliomas, leading to inconclusive diagnoses in approximately 7–15% of cases [44]. Despite being a minimally invasive procedure, SBB comes with inherent risk and complications, especially involving gliomas invading or originating in the brainstem, with complications occurring in 6% of patients [45]. Non-invasive radiogenomic analysis to identify glioma subtypes could not only offer prognostic value but also guide the use of targeted chemotherapies for complex and aggressive tumors, enhancing personalized treatment and possibly bettering patient outcomes. These non-invasive models could be particularly valuable in cases where surgical resection or tissue sampling is not feasible, either due to the tumor’s location, patient health concerns, or the patient’s decision to decline surgery. In such scenarios, radiogenomic models can offer prognostic insights, helping to guide the selection of appropriate radiotherapy and/or chemotherapy options. Moreover, radiomic models can be extremely useful in monitoring and predicting treatment responses in cases of unresectable or difficult-to-resect tumors such as H3 K27M-mutant diffuse midline gliomas [46, 47].

Structural MRI sequences are limited by the human eye’s capability to analyse visual data and often cannot differentiate between tumor recurrence, pseudoprogression, pseudoresponse, and radiation necrosis due to the complex nature of Gliomas [48, 49]. Radiomic and radiogenomic models aim to enhance our understanding of the genetic landscape of tumors through non-invasive imaging, while promising, their integration into clinical practice remains a work in progress. These models can prove to be particularly useful when a biopsy is challenging and molecular data is critical for management. For example, diffuse midline glioma, H3 K27-altered, has a significantly poor prognosis in comparison to the wild type, with 3-year overall survival of 5% and 2-year overall survival of less than 10% [50–53]. Prediction of H3 K27-altered status plays an essential role in tumor diagnosis, survival prediction, and therapeutic decision-making, and a few recent studies have found that an MRI-based radiomics signatures may significantly outperform predictions based on conventional MRI features [54, 55].

Even with rapid advancement in cancer care in the last decade, the prognosis of many CNS tumors remains poor. According to the latest CBTRUS 2023 report, the 5-year survival rate in patients diagnosed with malignant CNS tumors was only 35.7% [56], which has remained fairly stable in the last decade [57]. There is a large variation in median survival rate across different CNS tumor histopathologies, with glioblastoma having the lowest (8 months) and oligodendroglioma having the highest (199 months) [56]. Radiomic models can be potentially used to study the origin, extent, and overall architecture of tumors non-invasively, enhancing treatment monitoring and improving survival rates. Studies by Mauldin et al. [58]. and Sun et al. [59]. have highlighted the potential of using radiomic signatures to predict immunotherapy responses by assessing tumor infiltration by CD8 cells. While integrating immunotherapy into glioma treatment has been challenging, these findings might lead to new treatment avenues including vaccines, oncolytic viruses, immune checkpoint inhibitors, and genetically modified T cells [60, 61].

The field of computer vision is shifting from traditional semantic and radiomic methods to a combination of radiomics and deep learning-based feature extraction. This fusion of deep learning’s adaptable representational abilities with the interpretable characteristics of radiomics enables the acquisition of insightful knowledge in a data-centric fashion. This integrated approach, coined as Deep Radiomics employs neural networks to extract and analyse complex features from medical images automatically and streamlines the workflow, minimizes human error, and could provide more accurate diagnostics and treatment evaluations, leading to enhanced patient care [62]. Furthermore, radiomic models provide the benefit of analysing the entire tumor volume, overcoming the sampling limitations of tissue biopsies that may not be representative or might miss important information due to small sample yield [63]. This complete examination can lead to more precise diagnoses, improved prognostic assessments, and, as a result, better-informed clinical decision-making.

## Limitations

Radiomics and radiogenomics are rapidly advancing domains of medicine but have many obstacles to overcome before being established in standard patient care in CNS tumor management. As a nascent field, there is an absence of standardization of acquisition parameters and radiomic approaches. Many studies using retrospective data lack external validation, have incomplete results, or contain unidentifiable confounding variables in the source data. The culmination of these factors results in poor reproducibility secondary to variability and lack of consistency. To address these issues and standardize radiomics-specific reporting, Lambin et al. [64] proposed the concept of a Radiomics Quality Score (RQS) consisting of 16 key components, potentially enabling a holistic evaluation of both the reproducibility and reliability of results reported in Radiomic studies.

The limited availability of public databases with annotated radiological data is another obstacle to further validation and proliferation of Radiomic models. Small datasets with many variables can lead to overfitting, where a model performs well on training data but poorly on unseen data. This can be mitigated by using larger datasets or selecting robust variables for analysis. Park et al. evaluated 51 original Radiomics research articles in neuro-oncology with the RQS [64] and showed that only 29.4% performed external validation, with few studies discussing clinical utility and none conducting a phantom study or cost-effectiveness analysis [65].

Pre-operative MRI protocols vary widely across studies, making it difficult to compare results and validate models due to the lack of a publicly available, standardized dataset. Recent efforts to predict MGMT methylation in a diverse glioblastoma MRI dataset highlight the need for larger, more standardized datasets to overcome data heterogeneity and improve model generalizability [66]. Hence, the generalizability of DL/ML models remains a concern, given the large amount of heterogeneous data required for model training and validation [67]. Current, publicly available datasets often do not reflect the heterogeneity of data acquired in real clinical practice and frequently lead to the development of models that cannot generalize well to real-world scenarios. For instance, most current radiomic and deep learning frameworks are trained on datasets with a standardized set of MRI sequences available for each patient studied. These models cannot adapt to instances in which patients may not have a complete set of MRI sequences available for reasons including data corruption, MRI contrast sensitivity, etc. Several studies have begun to address this challenge via techniques including meta learning [68], data synthesis [69], and knowledge distillation [70], though future work still needs to be performed in order to develop models robust to the noisy data conditions inherent to real world implementation. Additionally, segmenting the Region of Interest (ROI) accurately is typically integral in radiogenomic pipelines, but variability in ROI selection can affect Radiomic features. Inconsistent ROI segmentation undermines Radiomic feature stability, and while new deep learning methods like U-net [71], V-net [72], UNet++ [73], and DeepMedic offer advancements, standardization is lacking.

Hence, multi-centric studies involving collaboration among research institutions are required to create professionally annotated standardized datasets for larger cohort studies, which can be split into training, testing, and validation datasets to reduce overfitting. This would also allow the researchers to test their algorithms on external cohorts and validate the robustness of their solutions. Federated learning, which facilitates multi-institutional validation of machine learning models without explicit data sharing using a distributed framework, can be used across multiple available centres in locations across the world, thus increasing the size and diversity of data used in training radiomic models.

Radiogenomics is a field that relies on establishing the association between a tumor’s phenotypic and genotypic nature. To make radiogenomic biomarkers reliable in oncology, standardization of assay criteria, image capture, segmentation, trial design, and analytical methods is imperative. Along with this, a focus on creating an extensive imaging database inclusive of genomic profiles, demographics, treatment details, and outcomes is essential. A task of this scale requires data sharing and collaboration among various institutions across the world. Initiatives like The Cancer Genome Atlas (TCGA) and The Cancer Imaging Archive (TCIA) demonstrate the benefits of shared, comprehensive genomic and imaging profiles [74, 75].

As newer and more advanced AI/Radiomics models continue to develop, ethical challenges also arise. The integration of AI into clinical practice introduces more ethical and regulatory issues. The US Food and Drug Administration (FDA) has strict regulations for computer-aided detection systems employing machine learning and pattern-recognition techniques. The introduction of AI/machine learning models poses novel regulatory challenges, demanding specialized guidance for submissions seeking approval [76]. AI models, unlike other intervention services, continue to evolve as they encounter more data; hence, testing at regular intervals of time is essential to ensure that their functionality meets the expected ethical standards.

While there is significant potential for the creation of diverse and heterogeneous datasets for healthcare AI, numerous technical and operational hurdles need to be addressed. Concerns regarding patient privacy, data ownership, intellectual property rights, and computation and storage limitations pose significant challenges. The complex web of regulatory policies differing across various geographical areas, and their ethical implications add another layer of complexity.

## Future directions

Recent developments in ML/DL techniques have significantly accelerated progress in neuroimaging and the application of radiogenomics to predict tumor genotypes. Deep learning-based tumor segmentation has shortened the arduous process of manual segmentation employed in a conventional radiomic approach. Combined Radiomics-CNN models hold immense promise for predicting the genotypic architecture of various CNS tumors, potentially revolutionizing personalized strategies in tumor treatment. However, rate-limiting steps such as the standardization of data interpretation, scarcity of publicly available imaging datasets, and the lack of large-scale, prospective clinical trials, remain before widespread clinical acceptance can be realized [66, 67, 77].

A major limitation across various studies analysed for this paper is lack of standardization of data interpretation. The Image Biomarker Standardisation Initiative (IBSI) is an independent international collaboration working towards the standardization of extraction of image biomarkers from acquired imaging for the purpose of high-throughput quantitative image analysis. Zwanenburg et al. [78] in a large scale multicentre, multiphasic study standardized a set of 169 radiomics features, thus enabling verification and calibration across different radiomics software. The IBSI workflow with standardization laid across multiple software packages can potentially improve the accuracy, reproducibility, and clinical utility of Radiomic features, ultimately paving the way for their integration into routine clinical practice. Datasets such as The Cancer Imaging Archive hosts imaging datasets of brain tumor collections (HGGs and LGGs), among other cancers, obtained from several institutions, have been widely used by the research community to develop and validate radiomics and radiogenomics tools [63]. Prestigious academic institutions and societies like the American Society of Neuroradiology (ASNR) and the Radiological Society of North America (RSNA) can play an important role in fostering radiomics and radiogenomics research as well as bridging the gap between promising studies and clinical applications.

Federated Learning (FL), in contrast to traditional centralized models, offers a solution by updating model parameters locally on users’ devices, with only the parameters being shared, not the data itself. This method maintains privacy and facilitates collaboration across geographically diverse areas, as demonstrated by Pati et al. [79] in their large-scale study on automated tumor boundary detection in Glioblastoma patients. When properly implemented with strict governance, standard data protocols, and clear clinical goals, FL has substantial potential to improve the performance of machine learning models in healthcare, making advanced models more accessible and equitable across diverse and resource-limited healthcare settings.

As noted in the WHO CNS-5 update, tumor genotype study is crucial for tumor classification and grading with significant implications for prognosis and treatment. In the realms of neurosurgery and neuro-oncology, radiomics/radiogenomic applications could transform the current paradigm by replacing lengthy and complex surgeries with rapid and crucial information delivery, thereby enhancing clinical decision-making and optimizing clinical outcome.

## Conclusion

WHO CNS-5 2021 update has emphasized the importance of tumor mutations and biomarkers in the classification and grading of gliomas. While radiogenomics holds promise as a non-invasive tool for assessing tumor genotypes and the tumor microenvironment, substantial research is needed to validate its predictive and prognostic capabilities before it can be effectively integrated into CNS tumor management. Recent advancements in radiomics and computer vision have propelled the field forward, with deep learning models and new imaging modalities now enabling rapid prediction of CNS tumor mutations. These innovations could potentially reduce the reliance on invasive surgeries, thereby minimizing associated risks and expediting treatment. Nevertheless, before radiogenomics can be fully integrated into clinical practice, several key issues must be addressed, including the standardization of data interpretation, scarcity of publicly available imaging datasets, and the lack of large-scale, prospective clinical trials. The evolution of machine learning and deep learning technologies is driving radiomics and radiogenomics toward a revolutionary role in neuroradiology and neuro-oncology, paving the way forward to improved diagnosis, prognostication, and ultimately personalized treatment approaches for brain tumor patients.

## Data Availability

No datasets were generated or analysed during the current study.

## Abbreviations

WHO: World Health Organization
CNS: Central Nervous System
MRI: Magnetic Resonance Imaging
MRS: Magnetic Resonance Spectroscopy
CT: Computed Tomography
PET: Positron Emission Tomography
HGG: High Grade Gliomas
LGG: Low Grade Gliomas
TDA: Topological Data Analysis
DLR: Deep Learning based Radiomics
VASARI: Visually AcceSAble Rembrandt Images
SBB: Stereotactic Brain Biopsy
TMZ: Temozolomide
IBSI: Image Biomarker Standardization Initiative
MGMT: O6-Methylguanine-DNA Methyltransferase
CDKN2A/B: Cyclin Dependent Kinase Inhibitor
SHH: activated-Sonic hedgehog-activated
TERTp: Telomerase Reverse Transcriptase promoter
2HG: 2-Hydroglutarate
IDH: Isocitrate Dehydrogenase
EGFR: Epidermal Growth Factor Receptor
rCBV: relative Cerebral blood volume
PSR: Permeability-surface area product (PSR)
