# Correction: New frontiers in domain-inspired radiomics and radiogenomics: increasing role of molecular diagnostics in CNS tumor classification and grading following WHO CNS-5 updates

**DOI:** 10.1186/s40644-024-00795-4

**Published:** 2024-10-31

**Authors:** Gagandeep Singh, Annie Singh, Joseph Bae, Sunil Manjila, Vadim Spektor, Prateek Prasanna, Angela Lignelli

**Affiliations:** 1 Neuroradiology Division, Columbia University Irving Medical Center, New York, NY USA; 2 Atal Bihari Vajpayee Institute of Medical Sciences, New Delhi, India; 3 Department of Biomedical Informatics, Stony Brook University, Stony Brook, USA; 4 Department of Neurological Surgery, Garden City Hospital, Garden City, MI USA

Following publication of the original article [1], we were notified that the affiliation of Prateek Prasanna was incorrect.

Originally published affiliation: Department of Neurological Surgery, Garden City Hospital, Garden City, MI, USA.

Correct affiliation: Department of Biomedical Informatics, Stony Brook University, Stony Brook, USA.

The original article has been corrected.
